# Protocol for bevacizumab purification using Ac-PHQGQHIGVSK-agarose

**DOI:** 10.1016/j.mex.2019.12.010

**Published:** 2019-12-16

**Authors:** Gabriela R. Barredo, Silvana L. Giudicessi, María C. Martínez Ceron, Soledad L. Saavedra, Santiago Rodríguez, Lucas Filgueira Risso, Rosa Erra-Balsells, Gustavo Mahler, Fernando Albericio, Osvaldo Cascone, Silvia A. Camperi

**Affiliations:** a Universidad de Buenos Aires, Facultad de Farmacia y Bioquímica, Cátedra de Biotecnología, Junín 956, 1113, Buenos Aires, Argentina; b CONICET-Universidad de Buenos Aires, Instituto de Nanobiotecnología (NANOBIOTEC), Facultad de Farmacia y Bioquímica, Junín 956, 1113, Buenos Aires, Argentina; c mAbxience SAU, Carlos Villate 5148, 1605, Munro, Buenos Aires, Argentina; d Universidad de Buenos Aires, Facultad de Ciencias Exactas y Naturales, Departamento de Química Orgánica, Pabellón II, Ciudad Universitaria, 1428, Buenos Aires, Argentina; e CONICET, Universidad de Buenos Aires, Centro de Investigación en Hidratos de Carbono (CIHIDECAR), Facultad de Ciencias Exactas y Naturales Pabellón II, Ciudad Universitaria, 1428, Buenos Aires, Argentina; f AGC Biologics, 22021 20th Avenue SE, Bothell, WA, 98021, USA; g School of Chemistry & Physics, University of KwaZulu-Natal, Durban, 4001, South Africa; h CIBER-BBN, Networking Centre on Bioengineering, Biomaterials and Nanomedicine, and Department of Organic Chemistry, University of Barcelona, 08028, Barcelona, Spain

**Keywords:** Bevacizumab purification using Ac-PHQGQHIGVSK-agarose, Peptide, Ligand, Downstream processing, Biopharmaceuticals, Monoclonal antibodies, Affinity chromatography, Solid phase peptide synthesis

## Abstract

Bevacizumab is a monoclonal antibody, produced in CHO cells, used for the treatment of many human cancers. It is an anti-vascular endothelial growth factor (antsi-VEGF) that blocks the growth of tumor blood vessels. Nowadays its purification is achieved by affinity chromatography (AC) using protein A which is a very expensive ligand. On the other hand, the peptide Ac-PHQGQHIGVSK contained in the VEGF fragment binds bevacizumab with high affinity. This short peptide ligand has higher stability and lower cost than protein A and it can be prepared very easily by solid phase peptide synthesis. The present protocol describes the synthesis of Ac-PHQGQHIGVSK-agarose and its use for affinity chromatography purification of bevacizumab from a clarified CHO cell culture.

• Ac-PHQGQHIGVSK-agarose capacity and selectivity are equivalent to those of protein A matrices.
• The peptide ligand shows a greater stability and lower cost. The lack of Trp, Met or Cys in the peptide ligand prevents its oxidation and extends the useful life of the chromatographic matrix.
• Mild conditions used during chromatography preserved the integrity of bevacizumab.

## Graphical abstract

**Figure:**
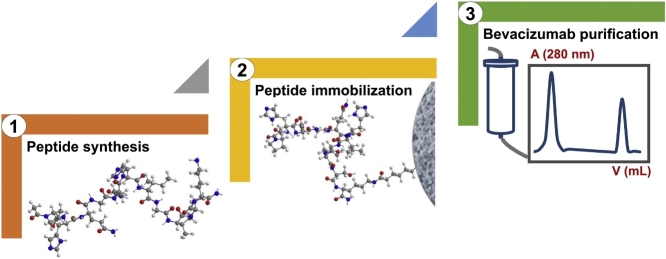

Specification Table

**Table:** 

Subject Area:	Biochemistry, Genetics and Molecular Biology
More specific subject area:	*Protein Purification*
Method name:	*Bevacizumab purification using Ac-PHQGQHIGVSK-agarose*
Name and reference of original method:	*Barredo GR, Giudicessi SL, Martínez Ceron MC, Saavedra SL, Rodriguez S, Filgueira Risso L, Erra-Balsells R, Mahler G, Albericio F, Cascone O, Camperi SA. 2020. A short peptide fragment of the vascular endothelial growth factor as a novel ligand for bevacizumab purification. Protein Expr Purif. doi: 10.1016/j.pep.2019.105500. Epub 2019 Sep 19.*
Resource availability:	*Not applicable*

## Method details

Bevacizumab (trade name: Avastin™) is an anti-vascular endothelial growth factor (anti-VEGF) that blocks the growth of tumor blood vessels. This monoclonal antibody (mAb), produced in CHO cells, is used for the treatment of many human cancers. Nowadays its purification is achieved by affinity chromatography (AC) using protein A, which is a high expensive ligand. Moreover, harsh elution conditions, that damage both the mAb and the protein ligand, are required to ensure bevacizumab recovery from the protein A affinity column. On the other hand, small peptides consisting of few amino acids represent ideal affinity chromatography ligands because they are much more physically and chemically stable than protein A and can be readily synthesized by standard chemistry in bulk amounts at a lower cost. Considering the bevacizumab binding site on the 85-Pro-His-Gln-Gly-Gln-His-Ile-Gly-92 VEGF segment [1], a peptide ligand for bevacizumab purification by AC was designed [2]. Val-Ser-Lys was introduced as a spacer arm to facilitate bevacizumab interaction with the immobilized ligand. Ligand site-directed immobilization on the agarose chromatographic support was ensured by the ε-amino group of the C-terminal Lys and the acetylation of the N-terminus. The short peptide designed, Ac-PHQGQHIGVSK-NH_2_, has higher stability and lower cost than protein A and it can be prepared very easily by solid phase peptide synthesis.

The present protocol describes the synthesis of the peptide Ac-PHQGQHIGVSK-NH_2_, its immobilization on agarose and the use of the Ac-PHQGQHIGVSK-agarose for affinity chromatography purification of bevacizumab from a clarified CHO cell culture.

The peptide ligand shows higher stability and lower cost than protein A. The lack of Trp, Met or Cys in the peptide ligand prevents its oxidation and extends the useful life of the chromatographic matrix. Bevacizumab binding to the peptidyl-agarose is achieved using as adsorption buffer 20 mM sodium phosphate, 1 M (NH_4_)_2_SO_4_, pH 7.0. Bevacizumab adsorption at high ionic strength suggests that the binding is largely hydrophobic. The elution is performed quantitatively by removing the (NH_4_)_2_SO_4_ from the running buffer, thus weakening the hydrophobic forces that supported the binding of bevacizumab to the chromatographic matrix. The mild elution conditions preserve the integrity of both the peptide ligand and the mAb. Furthermore, Ac-PHQGQHIGVSK-agarose capacity and selectivity are equivalent to those of protein A matrices.

### Step 1: Ac-PHQGQHIGVSK-NH_2_ synthesis

Material

• Fluorenylmethyloxycarbonyl (Fmoc) protected amino acids
• Rink-amide-MBHA resin (100–200 mesh, 0.67 meq/mg)
• *N,N*-dimethylformamide (DMF)
• CH_2_Cl_2_
• *N*-[(1 H-benzotriazol-1-yl)(dimethylamino)-methylene]-*N*-methylmethanaminium tetrafluoroborate *N*-oxide (TBTU)
• *N,N*-diisopropylethylamine (DIEA)
• Triisopropyl silane (TIS)
• 3,6-Dioxa-1,8-octanedithiol (DODT)
• Trifluoroacetic acid (TFA)
• Acetic anhydride (Ac_2_O)
• 20 % piperidine in DMF (v/v)
• TFA/TIS/H_2_O/DODT (92.5:2.5:2.5:2.5)
• Acetonitrile (MeCN)/H_2_O (1:1)
• Ethyl ether
• Orbital shaker
• Polypropylene column fitted with a polyethylene porous disk

Procedure

*Note:* Ac-PHQGQHIGVSK-NH_2_ is synthesized inside an efficient fume hood in polypropylene columns fitted with a polyethylene porous disk by solid phase Fmoc chemistry.

1 Place 1 g Rink-Amide-MBHA resin (0.67 meq/g) in a solid phase reactor.
  Note: The amount of resin to use depends on the amount of peptide to be synthesized. Considering an overall yield of 75 %, 1 g of resin is used to obtain 0.5 mol of peptide. The ligand Ac-PHQGQHIGVSK-NH_2_ is synthesized in enough amount to prepare the affinity chromatographic media. Usually, the purity of the crude peptide (>90 %) is enough for using it without further purification. In the case of a lower purity, a purification by reverse phase C_18_ liquid chromatography is carried out.
2 Wash the resin three times with CH_2_Cl_2_ and then with DMF.
3 Incubate with 20 % piperidine in DMF (v/v) (2 × 5 min) to remove the Fmoc group.
4 Wash the resin with DMF (5 × 1 min).
5 Weight the Fmoc-Lys(Boc)−OH (3 eq) and TBTU (3 eq) into a dry tube and dissolve them in a minimum amount of DMF.
6 Add the solution to the resin.
7 Add DIEA (4 eq) dropwise to the resin and mix.
8 Incubate the resin with agitation for 45 min at room temperature on an orbital shaker.
9 Wash the resin with DMF (2 × 1 mL) and CH_2_Cl_2_ (2 × 1 mL) by filtration or decantation.
10 To confirm the reaction coupling completion, test a small amount of resin (1−3 mg) with Kaiser test [3]. If positive, wash resin with DMF (2 × 1 min) and repeat coupling reaction with fresh reagents as indicated in steps 5-9. If negative, remove Fmoc group, wash the resin and couple the next Fmoc protected amino acid as indicated in steps 3–9.
11 After peptide elongation, acetylate terminal proline by adding Ac_2_O (10 eq.) and DIC (10 eq.) in enough CH_2_Cl_2_ to make the swollen resin just mobile to agitation.
12 Incubate during 1 h at room temperature.
13 To test the completion of the acetylation reaction, perform the chloranil test with a small amount or resin (1−3 mg) [4].
  *Note:* The Kaiser test is a qualitative test for primary amines; hence, it cannot be reliably applied to the evaluation of Pro acetylation. For secondary amines the chloranil test is recommended.
14 Remove side chain protecting groups and release the peptide from the resin by treatment with 15 mL of TFA/TIS/H_2_O/DODT (92:5.2:2.5:2.5) for 2 h.
15 Remove the resin by filtration, and add the filtrates containing the peptide to a 10-fold volume of cold ethyl ether to precipitate it.
16 Recover the precipitate by centrifugation (2000–3000 × *g* at 4 °C) during 10 min to ensure complete precipitation of the peptide.
17 Wash the peptide with extra cold diethylether and recover the peptide again by centrifugation.
18 Dissolve the peptide in MeCN/H_2_O (1:1) and lyophilize.

### Step 2: affinity matrix synthesis [5]

Material

• Dry *N*-hydroxysuccinimide (NHS)-activated agarose
• Dimethyl sulfoxide (DMSO)
• Peptide Ac-PHQGQHIGVSK-NH_2_
• Anhydrous triethylamine
• Ethanolamine
• Orbital shaker
• Polypropylene column fitted with a polyethylene porous disk

Procedure

*Note:* Ac-PHQGQHIGVSK-agarose is synthesized in polypropylene columns fitted with a polyethylene porous disk.

1 Place dry NHS-activated agarose (150 mg, that yields approximately 1 mL of hydrated resin) in a polypropylene column fitted with a polyethylene porous disk.
2 Wash the resin with pure DMSO (3 × 5 min).
3 Dissolve 50 mg of the peptide Ac-PHQGQHIGVSK-NH_2_ in 1 mL of DMSO and add to the NHS-agarose.
  Note: 50 mg of peptide Ac-PHQGQHIGVSK-NH_2_ is approximately 2-fold excess of the NHS group density in the agarose.
4 Add to the gel/ligand slurry an amount of anhydrous triethylamine equimolar to the amount of peptide and gently shake for 2 h at room temperature.
5 Filter the reaction mixture and save the filtrate for further analysis.
6 Wash the gel three times with DMSO.
7 Block any remaining unreacted groups by adding 50 μL of ethanolamine in 450 μL DMSO and then incubate for 30 min at room temperature.
8 Wash the matrix successively with DMSO, DMSO/H_2_O (70:30, 50:50 and 30:70) and finally with degassed deionized H_2_O.

### Step 3: evaluation of peptide immobilization on agarose

Material

• Absorption UV/VIS spectrophotometer
• 10 mm silica UV cell

Procedure

Peptide attachment is measured indirectly by quantifying the NHS released as a result of peptide immobilization.

1 Measure the absorbance at 260 nm of the filtrate saved in step 2 (item 5).
2 Calculate the NHS concentration, whose molar extinction coefficient (ε) is 9600 M^−1^ cm^−1^ at 260 nm [6].

### Step 4: Adsorption isotherms for bevacizumab binding to Ac-PHQGQHIGVSK-agarose

Material

• Absorption UV/VIS spectrophotometer
• 10 mm silica UV cell
• Orbital shaker
• 20 mM sodium phosphate, 1 M (NH_4_)_2_SO_4_, pH 7.0
• Labeled conical centrifuge micro tubes
• Labeled polypropylene columns fitted with a polyethylene porous disk.
• Pure bevacizumab solutions of known concentration
• Bradford reagent
• Thermomixer (Eppendorf)
• Sigma Plot software (http://www.sigmaplot.com/products/sigmaplot/sigmaplot-details.php)

Procedure

Note: Adsorption isotherms are measured in stirred batch systems

1 Equilibrate the affinity support, Ac-PHQGQHIGVSK-agarose, with 20 volumes of the adsorption buffer (20 mM sodium phosphate, 1 M (NH_4_)_2_SO_4_, pH 7.0) in a chromatographic column.
2 Add 50 μL of the chromatographic matrix to sequentially labeled conical centrifuge micro tubes, together with increasing volumes of pure bevacizumab solution and the amount of buffer necessary to reach a final volume of 1 mL.
  *Note*: To add the 50 μL of the chromatographic matrix to each tube, prepare a 1:1 suspension of the chromatographic matrix in adsorption buffer and while agitating measure 100 μL of the suspension with an automatic pipette with the tip cut at the end in order to increase its diameter.
3 Prepare another set of labeled tubes with the same volume of protein stock solution and buffer but without matrix.
4 Gently shake the tubes overnight at 25 °C in Thermomixer to enable the system to reach its equilibrium.
5 Separate the resin by filtration using labeled polypropylene columns fitted with a polyethylene porous disk.
6 Measure the protein concentration in the filtrate with Bradford reagent.
7 Determine free protein concentration at equilibrium (c*) with the first set of tubes and the total protein concentration at the beginning of the experiment (c_t_) with the second set of tubes.
8 Calculate the amount of bevacizumab bound to the immobilized peptide at equilibrium, per unit of total chromatographic matrix volume (q*), as:
  (1) q* = (c_t_-c*)1000/50
9 To determine the maximum adsorption capacity for bevacizumab per volume of chromatographic matrix (q_m_) and the dissociation constant (K_d_), non-lineal curve regression of the q* = f (c*) graph is performed with *Sigma Plot* software, using a one-to-one Langmuir binding mode [7]:
  (2) q* = q_m_·c*/(K_d_ + c*)

### Step 5: bevacizumab purification by peptide Ac-PHQGQHIGVSK-agarose affinity chromatography from the CHO cell culture

Material

• Adsorption buffer: 20 mM sodium phosphate, pH 7.0, 1 M (NH_4_)_2_SO_4_
• A column containing 0.5 mL (0.5 × 2.5 cm) of Ac-PHQGQHIGVSK-agarose
• Elution buffer: 20 mM sodium phosphate, pH 7.0
• Absorption UV/VIS spectrophotometer

Procedure

1 Clarify the CHO cell culture containing bevacizumab by centrifugation or filtration.
2 Add to the sample the amount of (NH_4_)_2_SO_4_ necessary to achieve a final concentration of 1 M.
3 Equilibrate the chromatographic column containing Ac-PHQGQHIGVSK-agarose with 20 volumes of adsorption buffer.
4 Load the column with CHO cell culture filtrate containing bevacizumab.
  Note: the amount of bevacizumab loaded to the column must be lower than the maximum capacity of the chromatographic matrix synthesized.
5 Wash the column with adsorption buffer until the absorbance at 280 nm achieves the baseline value.
6 Elute the bound protein by adding 20 mM sodium phosphate, pH 7.0.
7 Measure total protein concentration with Bradford reagent using pure bevacizumab as the protein standard [8].
8 Measure bevacizumab concentration by HPLC using a protein A analytical column as per Zou et al. [9].
9 Perform sodium dodecyl sulfate polyacrylamide gel electrophoresis (SDS-PAGE) (12.5 % under reductive conditions) as described by Laemmli [10] and stain gels with Coomassie Blue following the standard procedure.

## Method validation

Peptide Ac-PHQGQHIGVSK-NH_2_ is easy to obtain with high purity by solid phase peptide synthesis. Ligand site-directed immobilization on the NHS activated agarose is ensured by the ε-amino group of the C-terminal Lys and the acetylation of the N-terminus. The reaction is performed in organic solvent to prevent NHS ester hydrolysis, thus promoting higher immobilization yields [5]. When using Dry Pierce™ NHS-Activated Agarose, 17 μmol of peptide per mL of matrix was obtained with a maximum capacity of 38 mg of bevacizumab per mL of matrix. Similar results may be obtained using other NHS activated matrices such as NHS-activated Sepharose from GE Healthcare.

**Fig. 1:**
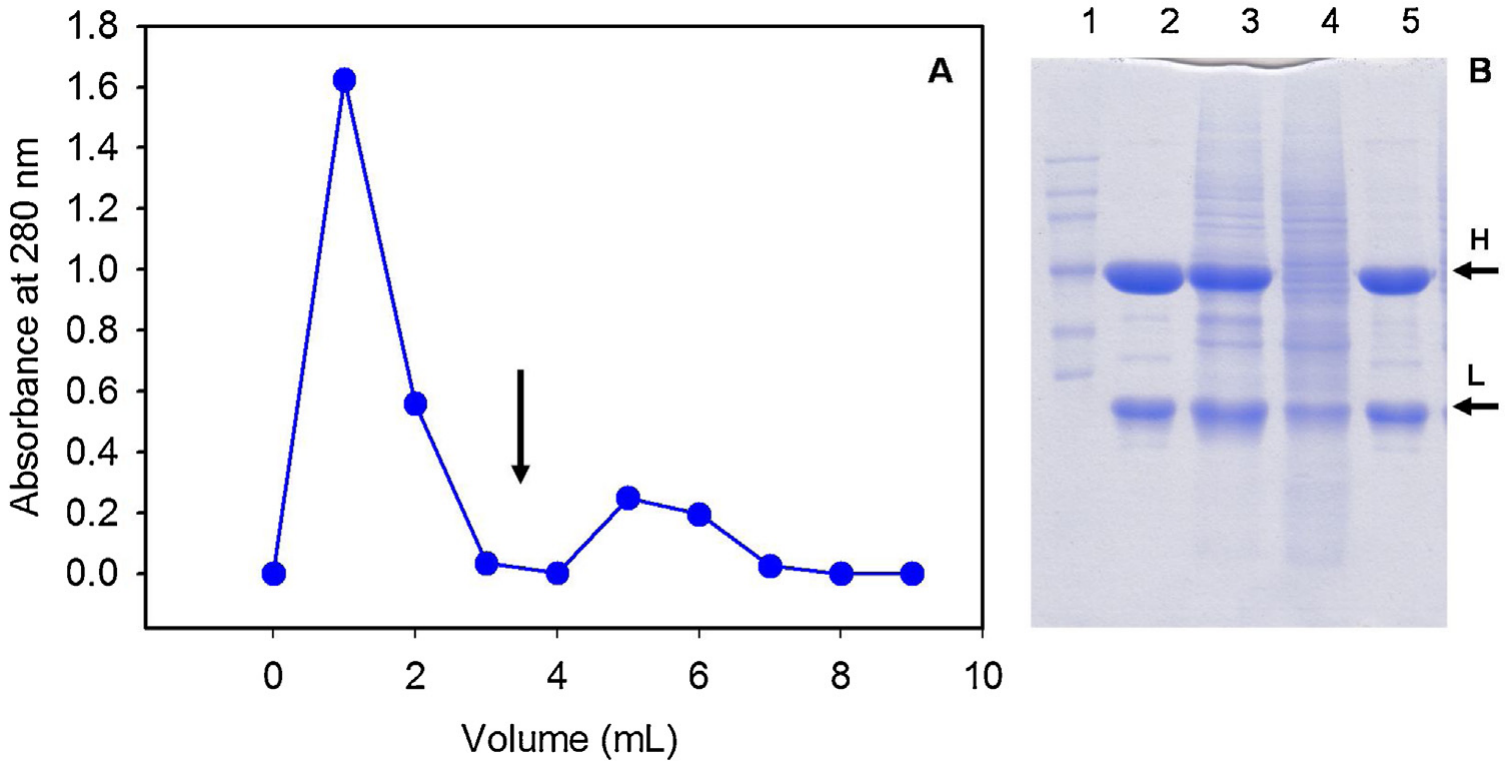
A) Bevacizumab purification from a CHO cell extract by affinity chromatography using Ac-PHQGQHIGVSK-agarose. The adsorption buffer was 20 mM sodium phosphate pH 7.0, 1 M (NH4)_2_SO_4_. The elution was performed with 20 mM sodium phosphate buffer, pH 7.0. The arrow indicates the buffer change. B) SDS-PAGE gels under reducing conditions of the chromatographic fractions. Lanes: 1) Protein molecular weight marker (MW); 2) Pure bevacizumab standard; 3) Bevacizumab-producing CHO cell filtrate; 4) and 5) washing and elution fractions, respectively.

Fig. 1 shows the profile, together with the corresponding SDS-PAGE of the chromatographic fractions, using 20 mM sodium phosphate, pH 7.0, 1 M (NH_4_)_2_SO_4_ as adsorption buffer and 20 mM sodium phosphate, pH 7.0 as elution buffer. The peptidyl-agarose adsorbs bevacizumab from the CHO cell culture filtrate while most contaminants pass through. Although the pass-through peak of the chromatogram is much higher than the elution peak, the protein concentration in both fractions are comparable. That is due to the contribution of the culture medium to the absorbance at 280 nm of the pass-through peak.

Table 1 shows the purification obtained using buffers 20 mM sodium phosphate, 1 M (NH_4_)_2_SO_4_, pH 7.0, and 20 mM sodium phosphate, pH 7.0, as adsorption and elution buffer respectively. The yield was 94 % and the purity 98 %.

**Table 1:** Purification chart of bevacizumab using Ac-PHQGQHIGVSK-agarose matrix.

Sample	bTotal protein (mg)	cTotal bevacizumab (mg)	dPurity %	Fold purification	Yield (%)
aCrude sample	0.487	0.219	45.0	1.0	100
Eluate fraction	0.210	0.206	98.1	2.2	94.1

a Bevacizumab-producing CHO cell culture filtrate from mAbxience SAU.

b Protein concentration determined by Bradford reagent.

c Bevacizumab concentration was determined by HPLC with a protein A column.

d Purity defined as amount of bevacizumab as a fraction of total amount of protein.

## Declaration of Competing Interest

The authors declare that they have no known competing financial interests or personal relationships that could have appeared to influence the work reported in this paper.
